# The GRADE approach for assessing new technologies as applied to apheresis devices in ulcerative colitis

**DOI:** 10.1186/1748-5908-5-48

**Published:** 2010-06-16

**Authors:** Nora Ibargoyen-Roteta, Iñaki Gutiérrez-Ibarluzea, Rosa Rico-Iturrioz, Marta López-Argumedo, Eva Reviriego-Rodrigo, Jose Luis Cabriada-Nuño, Holger J Schünemann

**Affiliations:** 1 Basque Office for Health Technology Assessment (Osteba), Department of Health and Consumer Affairs of the Basque Country, Vitoria-Gasteiz, Spain; 2 Department of Gastroenterology, Galdakao-Usansolo Hospital, Osakidetza (Basque Health Service), Galdakao, Spain; 3 Department of Clinical Epidemiology and Biostatistics, McMaster University, Hamilton, Canada

## Abstract

**Background:**

In the last few years, a new non-pharmacological treatment, termed apheresis, has been developed to lessen the burden of ulcerative colitis (UC). Several methods can be used to establish treatment recommendations, but over the last decade an informal collaboration group of guideline developers, methodologists, and clinicians has developed a more sensible and transparent approach known as the Grading of Recommendations, Assessment, Development and Evaluation (GRADE). GRADE has mainly been used in clinical practice guidelines and systematic reviews. The aim of the present study is to describe the use of this approach in the development of recommendations for a new health technology, and to analyse the strengths, weaknesses, opportunities, and threats found when doing so.

**Methods:**

A systematic review of the use of apheresis for UC treatment was performed in June 2004 and updated in May 2008. Two related clinical questions were selected, the outcomes of interest defined, and the quality of the evidence assessed. Finally, the overall quality of each question was taken into account to formulate recommendations following the GRADE approach. To evaluate this experience, a SWOT (strengths, weaknesses, opportunities and threats) analysis was performed to enable a comparison with our previous experience with the SIGN (Scottish Intercollegiate Guidelines Network) method.

**Results:**

Application of the GRADE approach allowed recommendations to be formulated and the method to be clarified and made more explicit and transparent. Two weak recommendations were proposed to answer to the formulated questions. Some challenges, such as the limited number of studies found for the new technology and the difficulties encountered when searching for the results for the selected outcomes, none of which are specific to GRADE, were identified. GRADE was considered to be a more time-consuming method, although it has the advantage of taking into account patient values when defining and grading the relevant outcomes, thereby avoiding any influence from literature precedents, which could be considered to be a strength of this method.

**Conclusions:**

The GRADE approach could be appropriate for making the recommendation development process for Health Technology Assessment (HTA) reports more explicit, especially with regard to new technologies.

## Background

### Ulcerative colitis and apheresis

Ulcerative colitis (UC) is a chronic disease of the colonic mucosa that is commonly treated with corticosteroid therapy to achieve clinical remission. Corticosteroids are used empirically in patients with moderate-to-severe UC despite the fact that relapse in patients who initially responded to these drugs is common. In addition, steroid therapy is associated with frequent side effects, especially when used for a long time [1]. In the last few years, a new non-pharmacological treatment, termed apheresis, has been reported to produce similar results to those obtained with corticosteroids in terms of disease remission [2-6].

Apheresis devices lower the elevated blood leukocyte and platelet levels found in active UC resulting from the activation behaviour and increased survival time of these blood components [7]. Leukocytapheresis (LCAP) and granulocytapheresis (GCAP) are the most frequently used apheresis treatments [8], which usually involve five sessions (one per week), although one or two sessions per week can be used for a period of time ranging from five to ten weeks [1,2,4,5,9,10]. However, the number of sessions can vary depending on the severity of the disease or the response to corticosteroid treatment, thus making the comparison of different studies somewhat difficult [11]. Hanai *et al*. reported that patients with severe active UC who were corticosteroid-naïve responded readily to granulocyte-monocyte apheresis (GMA), thereby avoiding steroid therapy [3]. These observations indicate that GMA might be a substitute for corticosteroid treatment in these patients, thereby allowing them to avoid the possible side effects of these drugs.

It has been reported that approximately 20% of patients with UC have a chronic active disease that often requires several courses of systemic steroids to achieve clinical remission. However, this treatment regime is often followed by relapse of symptoms during steroid tapering (continuous reduction of the dosage of corticosteroids once the initial high dosage has produced significant clinical improvement) or soon after their discontinuation.

Multiple studies have suggested that selective apheresis may be effective as a steroid-sparing treatment [12] because the resulting reduction in the peripheral levels of granulocytes and monocytes produced by GCAP might mitigate inflammation and delay relapse during steroid tapering in steroid-dependent patients [1].

### Recommendation development

When assessing a health technology, many methodologies have been used to establish recommendations based on existing systematic reviews or other study designs, including SIGN (Scottish Intercollegiate Guidelines Network) [13] and The Oxford Centre for Evidence-Based Medicine [14]. The GRADE (Grading of Recommendations Assessment, Development and Evaluation) approach has been developed by an informal collaboration group of guideline developers, clinicians, and methodologists with the aim of developing and disseminating a sensible and transparent approach to grading quality of evidence and strength of recommendations [15-17]. This approach is based on an assessment of other systems, including SIGN, and involves members from numerous international organizations. It was created to assess the quality of evidence and elaborate recommendations in clinical guidelines [15,18-22], therefore the application of this methodology would be of interest for health technology assessment (HTA) reports.

### Study objective

The objective of the present study was to use GRADE to develop recommendations regarding the use of apheresis devices for the treatment of UC, and to evaluate the strengths, weaknesses, opportunities, and threats found when using GRADE in this context in comparison with those found previously using the SIGN method.

## Methods

### Definition of the clinical questions

We selected two of the possible questions concerning the use of apheresis devices to treat UC using the PICO model (Patients, Intervention; Comparison and Outcomes) on the basis of two previously published documents [11,23]. These questions were as follows:

Question 1: Should apheresis devices be used to treat non-steroid-dependent or non-steroid-refractory UC patients to achieve clinical remission of the disease rather than standard corticosteroid treatment?

Question 2: Should apheresis devices be used as an adjunct treatment with corticosteroids to treat steroid-dependent UC patients with the aim of sparing or withdrawing corticosteroids rather than standard corticosteroid treatment?

### Definition and assessment of all Relevant Outcomes

Five researchers (NI-R, IG-I, RR-I, ML-A and ER-R) defined the outcomes of interest for each question based on prior work concerning the development of a monitoring system for measuring the effectiveness and safety of apheresis devices in UC patients [11]. The outcomes defined for the first question were: clinical remission one month after treatment (defined as Mayo Index ≤ 2) [24]; endoscopic remission one month after treatment (Endoscopic Mayo Subindex ≤ 1); and clinical remission 12 months after treatment. The following variables were defined to evaluate the safety of the treatment: percentage of patients with mild adverse events (those requiring continuing observation but no specific therapy) and percentage of patients with moderate to severe adverse events (with moderate events being defined as those requiring transient therapeutic countermeasures, but not interruption of therapy and severe events those resulting in sequelae or increased risk of death or requiring discontinuation of UC trial therapy). The outcomes defined for the second question were as follows: percentage of patients who do not require corticosteroids one month after the last apheresis session, mean reduction of corticosteroids dose one month after treatment, clinical remission one month after treatment (Mayo Index ≤ 2 and no corticosteroids), endoscopic remission one month after treatment (endoscopic subindex ≤ 1 and no corticosteroids), improvement of quality of life (as measured by the Inflammatory Bowel Disease Questionnaire, or IBDQ, which is able to distinguish between active UC disease and remission stage), colectomy rate during follow-up, percentage of patients with long-term side-effects of both treatments, and clinical remission maintained 12 months after treatment.

Each group member scored all defined outcomes from 1 to 9 (from least to most important). If major differences between individual scores were obtained, the relevance of that particular outcome was discussed to reach consensus. Critical outcomes were defined as those with a final score of between 7 and 9, and important outcomes as those with a final score of between 4 and 6 (See Table 1). Those outcomes scoring less than 4 points were not considered further.

**Table 1:** Assessment of the importance of the defined outcomes

Outcomes of interest for the first question	R1	R2	R3	R4	R5	Importance
1. Clinical remission one month after treatment	8	9	9	9	8	CRITICAL
2. Endoscopic remission one month after treatment	6	8	7	8	7	CRITICAL
3. Percentage of patients with mild adverse effects	8	6	7	6	5	IMPORTANT
4. Percentage of patients with moderate-to-severe adverse effects	9	8	9	8	8	CRITICAL
5. Clinical remission 12 months after treatment	7	6	8	6	8	IMPORTANT#

**Outcomes of interest for the second question**	**R1**	**R2**	**R3**	**R4**	**R5**	**Importance**

1. Percentage of patients who don't require corticosteroids one month after treatment	8	9	8	8	8	CRITICAL
2. Mean Reduction of Corticosteroids dose one month after treatment	9	6	7	6	6	IMPORTANT
3. Clinical remission one month after treatment (no corticosteroids)	8	8	7	8	8	CRITICAL
4. Endoscopic remission one month after treatment (no corticosteroids)	7	8	6	6	8	IMPORTANT^#^
5. Improvement of Quality of life (Inflammatory Bowel Disease Questionnaire, or IBDQ)	7	8	8	6	9	CRITICAL
6. Colectomy rate during the follow-up	9	9	9	8	9	CRITICAL
7. Percentage of patients with long-term adverse effects	9	8	8	8	9	CRITICAL
8. Clinical remission 12 months after treatment	7	6	7	7	8	CRITICAL

*R1: Researcher n1; R2: Researcher n2; R3: Researcher n3; R4: Researcher n4; R5: Researcher n5.

^# ^The group resolved by discussion that these outcomes should be considered as Important outcomes

### Literature search and study selection

A previous systematic review [25] was used to assess the use of apheresis devices in the treatment of UC. This research was updated by searching the following databases (up to May 2008): MEDLINE, Cochrane, EuroScan, INAHTA, ISI, Current Controlled Trials, National Guidelines Clearinghouse, New Zealand Guidelines group, SIGN, Fisterra, Lilacs, GETECCU, and the Cochrane-IBD (Inflammatory Bowel Disease) group. Boolean operators were used to combine free text such as 'inflammatory bowel disease', 'ulcerative colitis', 'Crohn's disease', 'apheresis', 'immunomodulation', 'leukocytapheresis', 'granulocytapheresis' and 'lymphocytapheresis' with controlled vocabulary. The results of this search were redefined using the Cochrane Collaboration's search filters to identify preferably randomized controlled clinical trials. We included studies if: the effectiveness of apheresis was assessed compared to conventional therapy; the safety of apheresis was evaluated; or the cost-effectiveness of the treatment was analysed. Case series with less than ten patients and studies with no control group were excluded [11].

### Assessment of the outcomes

The overall quality of the evidence for each outcome was assessed according to the considerations defined by the GRADE approach: study design limitations that may bias the estimates of the treatment effect; inconsistent results due to unexplained heterogeneity; indirectness of evidence because of indirect comparisons or indirect population, intervention, comparator, or outcomes; and imprecision of the included studies due to small sample size, number of events, or wide 95% confidence intervals. When possible, meta-analysis procedures using the RevMan v.5 program were used to pool the data found for the outcomes of interest. The information obtained for each proposed question was summarised using the GRADE profiler (GRADE Pro) v.3.2 program [26].

### Agreeing on recommendations

After assessing the evidence found for each outcome, the overall quality for each question was evaluated. The balance between risks and benefits was discussed, and the costs and patient values were taken into consideration when available. Finally, the recommendations provided to the decision makers were graded and defined by the group on the basis of all judgements made.

The overall process was reviewed by one of the members of the GRADE working group (HJS), who supervised and resolved any doubts concerning the methodological aspects of the process. JLC-N, a gastroenterologist and expert in inflammatory bowel disease (IBD) who had previous experience with the assessed treatment, reviewed the PICO questions and possible outcomes for each defined question.

### SWOT analysis to evaluate the use of the GRADE approach for assessing new technologies

A SWOT (Strengths, Opportunities, Weaknesses and Threats) analysis was performed to evaluate our use of the GRADE approach to establish recommendations concerning this new technology. Strengths were defined as those attributes of the GRADE approach that were helpful for achieving the objective, and weaknesses as those attributes considered detrimental for this purpose. Opportunities were defined as those external conditions considered helpful for achieving the objective, and threats as those external conditions which could be detrimental to the objective.

The group of researchers that developed this work informally discussed the likely strengths, opportunities, weaknesses, and threats found when using the GRADE approach in this context. HJS did not participate in this activity because of his role in the development of GRADE. An evaluation was performed by each researcher involved (NI-R, IG-I, RR-I, ML-A, ER-R), and all the issues identified were summarized and discussed to develop common themes. Each researcher scored all of the items from 1 (least important) to 9 (most important). Finally, the total and median scores for each issue identified were calculated and used to order these issues by importance.

## Results

### Results for the first question

'Should apheresis devices be used to treat non-steroid-dependent or non-steroid-refractory UC patients to achieve clinical remission of the disease rather than standard corticosteroid treatment?'

The consensus reached concerning the relative importance of the outcomes defined for this question is presented in Table 1. The controlled clinical studies reported by Nishioka [5], Hanai [4], and Bresci [2] were included. The patients studied by Sands [27] were not defined in terms of the clinical scenarios considered in this study; therefore this trial was not included in the analysis.

**Figure 1:**
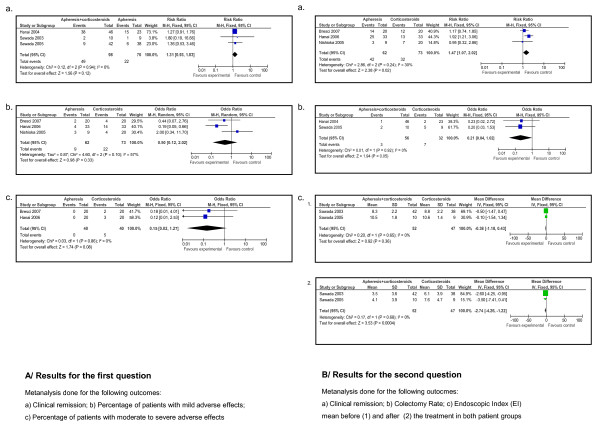
**Meta-analysis performed for the outcomes related to each proposed clinical question**.

Table 2 summarizes the information found for each outcome in a GRADE profile. When two or more studies were found for the same outcome, the data were meta-analyzed (Figure 1A).

**Table 2:** GRADE evidence Profile for the first clinical question

Quality assessment							Summary of findings					Importance
								
							No of patients		Effect		Quality	
		
No of studies	Design	Limitations	Inconsistency	Indirectness	Imprecision	Other considerations	Apheresis systems	Corticosteroid treatment	Relative (95% CI)	Absolute		
**Clinical Remission one month after treatment (follow-up median 3 weeks)**												

3	randomised trial	serious^1^	no serious inconsistency^2^	no serious indirectness^3^	serious^4^	none	42/62 (67.7%)	32/73 (43.8%)	RR 1.47 (1,07 to 2,02)	206 more per 1000 (from 31 fewer to 447 more)	⊕⊕OO LOW	CRITICAL

**Endoscopic Remission one month after treatment (follow-up mean 1 weeks; range of scores: 0-0; Better indicated by less)**												

1	randomised trial	serious^5^	no serious inconsistency^6^	serious^7^	serious^4,6^	none	20	20	-^8^	-^8^	⊕OOO VERY LOW	CRITICAL

**Mild adverse effects of the treatment (follow-up median 3 weeks)**												

3	randomised trial	serious^1^	serious^9^	no serious indirectness	serious^4,10^	none	9/62 (14.5%)	22/73 (30.1%)	OR 0.50 (0.12 to 2.02)	135 fewer per 1000 (from 258 fewer to 209 more)	⊕OOO VERY LOW	IMPORTANT

**Moderate to severe adverse effects (follow-up median 3 weeks)**												

2	randomised trial	serious^11^	no serious inconsistency	no serious indirectness	serious^4^	none	0/29 (0%)	5/40 (12.5%)	OR 0.15 (0.02 to 1.27)	105 fewer per 1000 (from 122 fewer to 30 more)	⊕⊕OO LOW	CRITICAL

**Clinical Remission 12 months after treatment - not measured**												

0	-	-	-	-	-	-	-	-	-	-		IMPORTANT

^1^We don't know if these studies are blinded. In the study of Nishioka et al, patients were free to choose the treatment they wanted to receive (it was not really a randomized controlled trial); nevertheless, it was included in the meta-analysis, although the quality was downgraded here. One of the studies (Hanai et al, 2006) was an abstract, although we found the information we needed.

^2 ^The percentage of patients in remission was not consistent in the included studies, although we made a meta-analysis and found that the p for heterogeneity was 0.24. Therefore, we did not consider this issue important enough for downgrading.

^3^The clinical remission was defined as a Mayo Index from 0 to 2 points, but in the included studies, the CAI was used, sometimes in combination with the EI (Endoscopic Index).

^4^There is a small number of patients and a very small number of events (less than 300).

^5^This RCT is not blinded.

^6^There is only one study.

^7^There were no data about endoscopic remission, but there was information about the Mean Endoscopic Index for the intervention and control group, before and after the treatment. After the treatment, the mean EI fell 5.5 and 6 points for the apheresis and the corticosteroids groups respectively.

^8^We couldn't calculate it, but it seems that there is no difference between treatments.

^9^The results obtained by Nishioka et al, 2005, are not consistent with the other two included studies. In the meta-analysis, we found an I2 of 57% and we considered that this heterogeneity was important enough to downgrade.

^10^95% CI for the total effect is wide.

^11^We don't know if included studies are blinded.

Some factors, such as the different definitions of clinical remission in the studies selected, complicated the analysis of the results. Although we used the Mayo Clinical Index to define clinical and endoscopic remission, a large number of different indices and definitions can be used for the same purpose [28], as was the case in some of these studies [1,2,5]. We found that the Rachmilewich Endoscopic Index (EI) was most often used to define endoscopic remission, although this outcome was not measured in the included studies. Some of these studies did, however, report the mean EI before and after treatment, therefore we used this outcome as an indirect measure of endoscopic remission (Table 2). We judged this indirectness to be serious enough to merit a further downgrade.

The findings for the first question can be summarised as follows: 

Balance between risk and benefits: there appears to be no difference in efficacy between apheresis devices and corticosteroids, both of which induce clinical remission in both non-steroid-dependent and non-steroid-refractory patients one month after treatment, although the effect of apheresis treatment is slower than that of corticosteroids in those patients that respond to them. The incidence of adverse effects with LCAP seems to be significantly lower than with high-dose corticosteroids, although these effects are generally transient and, in most cases, disappear during or shortly after the LCAP sessions [29]. The adverse effects of a short course of corticosteroids do not appear to be important. 

Remarks: the balance between risks and benefits is uncertain, although, in contrast to corticosteroids, apheresis treatment appears to be associated with more benefits than risks. The general quality of the evidence found to answer the clinical question was very low (see Table 2), although this treatment appears to have similar remission rates to corticosteroid therapy. Apheresis devices do not however seem to offer sufficient net benefit in terms of lower costs and more rapid effect than corticosteroids (in patients who respond to them) in this clinical context. Acute course and tapering of prednisone treatment cost was estimated at 218.3 euros, and the cost for Adacolumn^® ^treatment at 6,500 euros [30].

In conclusion, in light of the limited adverse effects of a two-month course of corticosteroids and their faster induction of remission and notably lower price, apheresis devices are unlikely to be of greater benefit than corticosteroid treatment in this context.

The panel therefore agreed on the following recommendation:

'For non-steroid-dependent and non-steroid-refractory UC patients, we recommend administration of corticosteroids rather than apheresis devices (GCAP or LCAP); weak treatment recommendation, very low quality of evidence.'

### Results for the second question

'Should apheresis devices be used as an adjunct treatment with corticosteroids to treat steroid-dependent UC patients with the aim of sparing or withdrawing corticosteroids rather than standard corticosteroid treatment?'

Table 1 shows the scores for the relative importance for each defined outcome. The studies by Hanai [1] and Sawada [10,29] were selected for this question. The retrospective study of Jo *et al*. [31] was excluded because the authors stated that compared groups were probably different (apheresis treatment was more likely to have been applied to patients resistant to or dependent on prednisolone).

Table 3 shows the GRADE profile obtained for the second question. A meta-analysis of the data was performed for the following outcomes: clinical and endoscopic remission, and the reduction of the dose of corticosteroids before and after treatment (Figure 1B).

**Table 3:** GRADE evidence Profile for the second clinical question.

Quality assessment							Summary of findings					Importance
								
							No of patients		Effect		Quality	
		
No of studies	Design	Limitations	Inconsistency	Indirectness	Imprecision	Other considerations	Apheresis systems plus Corticosteroid treatment	Corticosteroid treatment	Relative (95% CI)	Absolute		
**Percentage of patients wihtout corticosteroids one month after treatment (follow-up mean 14 weeks)**												

1	randomised trial	no serious limitations^1^	no serious inconsistency	no serious indirectness	serious^2,3^	none	10/46 (21.7%)	3/23 (13%)	OR 1.85 (0.46 to 7.52)	91 more per 1000 (from 67 fewer to 454 more)	⊕⊕⊕O MODERATE	CRITICAL

**Mean reduction of corticosteroid dose (follow-up median 7.5)**												

2	randomised trial	serious^4^	serious^5^	no serious indirectness	serious^3^	none	55	33	-	not pooled	⊕OOO VERY LOW	IMPORTANT

**Clinical remission one month after (follow-up median 1 weeks)**												

3	randomised trial	serious^4^	no serious inconsistency^6^	serious	serious^3^	none	49/98 (50%)	22/70 (31.4%)	RR 1.31 (0.93 to 1.83)	97 more per 1000 (from 22 fewer to 261 more)	⊕OOO VERY LOW	CRITICAL

**Endoscopic remission one month after treatment (follow-up median 1 weeks)**												

2	randomised trial	serious^4^	no serious inconsistency	serious^7,8^	serious^3^	none	52	47	-	-	⊕OOO VERY LOW	IMPORTANT

**Colectomy rate in the follow up (follow-up median 2 weeks)**												

2	randomised trial	serious^4^	no serious inconsistency	no serious indirectness	serious^3^	none	3/56 (5.4%)	7/19 (36.8%)	OR 0.21 (0.04 to 1.02)	275 fewer per 1000 (from 350 fewer to 6 more)	⊕⊕OO LOW	CRITICAL

**Quality of life improvement - not measured**												

0	-	-	-	-	-	-	-	-	-	-		CRITICAL

**Long term side effects - not measured**												

0	-	-	-	-	-	-	-	-	-	-		CRITICAL

**Clinical remission 12 months after treatment (follow-up mean 3.5 months)**												

1	randomised trial	no serious limitations	no serious inconsistency	serious^9^	serious^2,3^	none	39/46 (84.8%)	16/23 (69.6%)	RR 1.22 (0.9 to 1.36)	153 more per 1000 (from 70 fewer to 251 more)	⊕⊕OO LOW	CRITICAL

^1^Information about the randomization process is not provided in the main text, although it's pointed out that each patient was blindly assessed.

^2^There is only one study.

^3^Small sample size and less than 300 events.

^4^Patients included in selected studies could be different because the criteria used to define their steroid-response pattern were not the same. This was considered a limitation and, as a consequence, the quality was downgraded.

^5^The patients treated with apheresis and corticosteroids do not take the same dose of corticosteroids at the beginning. moreover, the pointing time when 'final' corticosteroid dose is measured is not the same in both studies (this is why we did not pool the data).

^6^In Sawada's both studies, clinical remission was more strictly defined (CAI = 0). nevertheless, consistency among results was found (see Figure 1B).

^7^They do not assess the Endoscopic remission as defined, but give data about the Mean EI before and after the treatment for both groups. we could pool the Mean EI before and after the treatment and compare them (See Figure 1B). Before the treatment, the mean EI was similar between groups, but after the treatment, the mean EI was almost 3 points lower for the apheresis plus corticosteroids group than for the corticosteroids group.

^8^None of the studies defined endoscopic remission taken into account the withdrawal of corticosteroids.

^9^In this study, the mean follow-up was 3.5 months, and not 12 months as defined for the outcome of interest

The length of follow up, the different indices used to define clinical and endoscopic remission and the lack of results for some of the selected outcomes complicated the assessment. Nevertheless, no differences in terms of clinical remission when using different protocols have been described in the literature [9,32].

The findings for the second question can therefore be summarised as follows: 

Balance between risks and benefits: Apheresis devices appear to be associated with more benefits than risks. As apheresis could mean that many patients with moderately active UC are spared corticosteroid therapy [1], the apparent risks of apheresis should be compared with the risks of receiving continuous corticosteroid treatment. 

Remarks: In this case, the balance between risks and benefits is uncertain and only very low-quality evidence was available to answer the question. Indeed, we were only able to find a single study assessing the cost of moderate-to severe UC in two scenarios: traditional treatment versus alternative treatment incorporating GCAP [30]. This study showed that the incorporation of GCAP into the therapeutic management of moderate-to-severe steroid-dependent UC patients is cost-effective and results in savings related to the reduction of adverse effects derived from corticosteroid use and a decreased number of surgical interventions. With regard to the patients' values and preferences, we found that some UC patients refused to be treated with corticosteroids [29,33]. Moreover, in a recent study of Crohn's disease patients' preferences, it was found that patients indicated a systematic preference for treatments that, amongst other issues, avoided the need for steroids [34].

Thus, the panel agreed to make the following recommendation:

'We recommend that patients with steroid-dependent UC should be treated with apheresis devices (GCAP or LCAP) together with corticosteroids to help them reduce or withdraw continuous corticosteroids intake (weak treatment recommendation, very low quality of evidence)'

### SWOT analysis to evaluate whether the GRADE approach is appropriate for assessing new technologies

The SWOT analysis results regarding the suitability of the GRADE approach for assessing new technologies are presented in Table 4.

**Table 4:** SWOT analysis results

Strengths	Median (total score)	Weaknesses	Median (total score)
**S1: **Elaboration and grading of the recommendations starts at the beginning of the process when ranking the importance of the outcomes	**8** **(39 points)**	**W1: **Time-consuming method	**8** **(38 points)**
**S2: **Patients' values are considered to define and grade the outcomes, thus avoiding the influence of literature results	**8** **(38 points)**	**W2: **The strength of recommendations does not only depend on the quality of the evidence found	**6** **(33 points)**
**S3: **Patients' opinions taken into account during the process	**7** **(37 points)**	**W3: **Requires academic training to understand how it works	**7** **(31 points)**
**S4: **Explicit assessment of the quality of outcomes across studies	**7** **(35 points)**	**W4: **Some elements continue to be developed	**5** **(29 points)**
**S5: **Individual analysis of the outcomes, taking into account the 'effect' and applicability aspects during elaboration of the recommendations	**7** **(34 points)**		
**S6: **Collaboration from the beginning facilitates the acceptance of results	**7** **(33 points)**		

**Opportunities**	**Median (total score)**	**Threats**	**Median (total score)**

**O1: **Possibility to use in HTA, including new technologies, due to its transparency and systematic methodology	**8** **(38 points)**	**T1: **Difficulties with new technologies: low number of studies, heterogeneity, unsuitable outcomes...	**6** **(34 points)**
**O2: **Identifies outcomes to be considered in future research	**7** **(37 points)**	**T2: **Complexity of the method can limit its use by experts	**7** **(34 points)**
**O3: **Developed software that helps the process	**7** **(37 points)**	**T3: **Lack of institutional support	**6** **(25 points)**

The most relevant strength found was that the elaboration and grading of the quality of evidence and recommendations starts at the very beginning of the process with the definition and importance rating of the outcomes for the proposed clinical question. The GRADE approach also takes into account the patients' values and avoids the influence of any outcomes reported in the literature. In contrast, application of the GRADE approach was considered to be more time-consuming than other methods such as SIGN because information has to be sought for all defined outcomes. Despite this, we consider that using the GRADE approach in HTA, including new technologies, could be beneficial due to its transparency and systematic methodology.

## Discussion

The development of recommendations in healthcare has always been problematic, and many different methods have been used [13,14]. Over the last two years, our group has been working on the introduction of the GRADE approach in the Spanish context because this approach incorporates the advantages of prior methods and continues to integrate new developments in health research methodology [15,18-22,35,36]. The aim of the current study was to apply this approach in a different context from the development of typical clinical practice guidelines, specifically the assessment of new and emerging health technologies, and for this purpose we chose the case of apheresis devices in UC treatment.

As a limitation of this study, we should note here that we did not perform a controlled study comparing the GRADE approach with another method for evaluating the quality of evidence and strength of recommendations, which would have been of interest in order to validate/confirm our results in an objective manner. Nevertheless, to learn from this study and draw conclusions about our experience, we performed a SWOT analysis to analyze the strengths, weaknesses, opportunities, and threats found when using the GRADE approach in this context.

We should also point out that our results may be influenced by our relatively limited experience with using the GRADE approach. Indeed, our interpretation may have been influenced by the impression of the participants at several workshops we have run concerning the correct use of the GRADE approach, who declared it to be a more complicated method, particularly for clinicians, and more time consuming than other systems commonly used to elaborate clinical guidelines [37]. However, using the software and support material provided by GRADE may facilitate the production of evidence profiles and enhance transparency when formulating recommendations, as pointed out in our SWOT analysis (Table 4, opportunity 3).

The inclusion of only one clinical expert could also be a limitation of this study as having only 'one point of view' could bias our work. This study was based on a previous one undertaken in collaboration with four experts in IBD, therefore the role of the clinical expert in the current study was simply to resolve any doubts that may have arisen related to this disease. For a future controlled trial, it would be advisable to include more clinical experts to cover possible different points of view.

Another limitation of this study, which is not specific to GRADE, concerns the difficulties encountered in finding data for some relevant outcomes that were not measured or reported. This was the case for the outcome 'improvement of UC patients' quality of life', for which the IBDQ questionnaire is frequently used. This outcome was defined as critical for the second question, although it was not measured in any of the included studies. Similarly, despite recent reports showing that GMA seems to be effective long-term [38,39], no direct data were available for the outcome 'clinical remission after 12 months follow-up'. A similar situation was found for the definition of clinical remission in steroid-dependency, with some experts considering that this should be accompanied by complete withdrawal of steroids [33,38,40,41]. Our inability to locate these data made the assessment of the evidence more challenging. However, in the case of new technologies, the conclusions obtained upon application of the GRADE approach should help to ensure the correct definition of the outcomes of interest, which should then be evaluated in future research (Table 4, opportunity 2).

With regard to the strengths of this study, previous work by GETECCU (The Spanish Group for the study of Crohn's disease and UC) group members facilitated the definition of the outcomes of interest, which could facilitate the acceptance of final recommendations by clinicians (Table 4, strength 6). A qualitative study performed after a training course concerning the GRADE approach in Spain found that this approach was perceived to be more sensitive to the issues faced by professionals in practice [37] because the relevant outcomes are defined taking into account those outcomes considered to be important by both professionals and patients rather than on the basis of literature findings (Table 4, strength 2). As a consequence, the elaboration of recommendations starts at the very onset of the process on the basis of patients' values and important outcomes (Table 4, strength 1). We also attempted to take patients' values and preferences into account, which is a key strength of this method (Table 4, strength 3).

With regard to the clinical questions selected, the literature studies found indicate that selective leukocyte apheresis effectively removes activated granulocytes and monocytes/macrophages from the peripheral blood of UC patients while maintaining an excellent safety profile [42]. Indeed, some studies have proposed the use of apheresis devices as a first-line treatment for UC patients rather than corticosteroid therapy [3], and others have produced evidence regarding the efficacy of selective apheresis as a steroid-sparing treatment [12], which explains why these particular clinical questions were formulated. Other questions related to the use of apheresis devices in the treatment of UC could be proposed, such as the possible use of apheresis treatment for paediatric patients or patients with toxicity to corticosteroids.

The most challenging part of this study was the assessment of the evidence found for each outcome, partly due to the context of the disease and the characteristics of the new technology being assessed (Table 4, threat 1). Whereas the SWOT analysis suggested that the method was time consuming (Table 4, weakness 1) and required some academic training (Table 4, weakness 3), both of which could be considered a limitation for its use (Table 4, threat 2), evidence assessment is, in general, complicated irrespective of whether GRADE or other methods are used. It is therefore possible that other methods could be more time consuming if they are expected to produce similarly transparent results. Moreover, the GRADE approach offers the possibility of making explicit judgements about the consistency, indirectness, and precision of the results, which is considered to be beneficial when applied to new and emerging technologies (Table 4, opportunity 1 and strength 4).

We considered that the overall quality of the evidence for each question should be based on the critical outcome with the lowest quality of evidence. In our case, this quality was very low for both questions. As we have stated in the SWOT analysis, the GRADE approach judges the relative importance of different outcomes and their trade-offs, as well as the quality of evidence, explicitly rather than implicitly [35], which in our opinion facilitates the discussion and clarification of these judgements.

As we have mentioned before, although we consider that the information obtained from the SWOT analysis concerning the feasibility of using the GRADE approach in this context is useful, we also think that a controlled trial should be designed to study whether the recommendations made differ when using different methodologies for this purpose. This would give more detailed information regarding the utility of the GRADE approach in this context.

## Summary

Our study suggests that the GRADE approach could be an appropriate means of making the recommendation-formulation stage a more transparent part of the overall process of producing HTA reports. Such reports are especially relevant in the case of new technologies, although we expect that most such assessments would lead to weak recommendations due to the lack of information that accompanies the introduction of new health technologies. However, we also consider that this approach would help to determine what future research should take into account when new technologies are assessed. Furthermore, more studies should be conducted to develop the best approaches to making recommendations about new health technologies.

## Competing interests

We declare that one of the authors (HJS) works in the development of the GRADE approach, although his contribution to this study mainly involved teaching the other group members about GRADE, revising and discussing the study results, and helping the other group members to apply the GRADE approach correctly.

## Authors' contributions

Five authors (NI-R, IG-I, RR-I, ML-A and ER-R) participated in the whole process (application of the GRADE approach and performance of the SWOT analysis). HJS helped with the correct application of the GRADE approach in this context and during the revision and discussion of the results obtained for both processes. JLC-N gave advice and help concerning aspects related to the disease and the treatment considered. NI-R is its guarantor. All authors read and approved the manuscript.

## References

[B1] Hanai H Watanabe F Yamada M Sato Y Takeuchi K Iida T Tozawa K Tanaka T Maruyama Y Matsushita I Iwaoka Y Kikuch K Saniabadi AR Adsorptive granulocyte and monocyte apheresis versus prednisolone in patients with corticosteroid-dependent moderately severe ulcerative colitis Digestion 2004 70 1 36 44 10.1159/000080079 15297776

[B2] Bresci G Parisi G Mazzoni A Scatena F Capria A Treatment of patients with acute ulcerative colitis: conventional corticosteroid therapy (MP) versus granulocytapheresis (GMA): a pilot study Dig Liver Dis 2007 39 5 430 434 10.1016/j.dld.2007.01.001 17379588

[B3] Hanai H Watanabe F Takeuchi K Saniabadi A Bjamasson I Leukocyte adsorptive apheresis for the treatment of active ulcerative colitis: A prospective uncontrolled study Clin Gastroenterol Hepatol 2003 1 28 35 10.1053/jcgh.2003.50005 15017514

[B4] Hanai H Iida T Watanabe F Yamada M Takeuchi K Kikuyama M Maruyama Y Iwaoka Y Hirayama K Saniabadi AR Intensive granulocyte and monocyte apheresis versus intravenous prednisolone in patients with severe ulcerative colitis: a multicentre randomised conrolled study [abstract] Gut 2006 55 Suppl 2 A116531518

[B5] Nishioka C Aoyama N Maekawa S Shirasaka D Nakahara T Tamura T Fukagawa M Umezu M Abe T Kasuga M Leukocytapheresis therapy for steroid-naive patients with active ulcerative colitis: its clinical efficacy and adverse effects compared with those of conventional steroid therapy J Gastroenterol Hepatol 2005 20 10 1567 1571 10.1111/j.1440-1746.2005.03907.x 16174075

[B6] Yamamoto T Umegae S Kitagawa T Yasuda Y Yamada Y Takahashi D Mukumoto M Nishimura N Yasue K Matsumoto K Granulocyte and monocyte adsorptive apheresis in the treatment of active distal ulcerative colitis: a prospective pilot study Aliment Pharmacol Ther 2004 20 7 783 792 10.1111/j.1365-2036.2004.02189.x 15379839

[B7] McCarthy DA Rampton DS Liu YC Peripheral blood neutrophils in inflammatory bowel disease: morphological evidence of in vivo activation in active disease Clin Exp Immunol 1991 86 489 493 1684141 10.1111/j.1365-2249.1991.tb02958.x PMC1554212

[B8] Pineda AA Developments in the apheresis procedure for the treatment of inflammatory bowel disease Inflamm Bowel Dis 2006 12 Suppl 1 S10 S14 10.1097/01.MIB.0000195386.19268.b3 16378005

[B9] Sakuraba A Sato T Naganuma M Morohoshi Y Matsuoka K Inoue N Takaishi H Ogata H Iwao Y Hibi T A pilot open-labeled prospective randomized study between weekly and intensive treatment of granulocyte and monocyte adsorption apheresis for active ulcerative colitis J Gastroenterol 2008 43 51 56 10.1007/s00535-007-2129-6 18297436

[B10] Sawada K Kusugami K Suzuki Y Bamba T Munakata A Hibi T Shimoyama T Leukocytapheresis in ulcerative colitis: results of a multicenter double-blind prospective case-control study with sham apheresis as placebo treatment Am J Gastroenterol 2005 100 6 1362 1369 10.1111/j.1572-0241.2005.41089.x 15929771

[B11] Ibargoyen-Roteta N Gutiérrez-Ibarluzea I Cabriada-Nuño JL Clofent-Vilaplana J Ginard-Vicens D Domènech-Morral E Oliva-Oliva G Queiro-Verdes T Establecimiento de Estándares, registro y análisis de casos de tratamiento de la colitis ulcerosa mediante sistemas de aféresis Informes de Evaluación de Tecnologías Sanitarias 2006 Madrid: Plan Nacional para el SNS del MSC. Servicio de Evaluaciòn de Tecnologías Sanitarioas del País Vasco OSTEBA N° 2006/5

[B12] Bianchi Porro G Cassinotti A Ferrara E Maconi G Ardizzone S Review article: the management of steroid dependency in ulcerative colitis Aliment Pharmacol Ther 2007 26 779 794 17767462 10.1111/j.1365-2036.2007.03334.x

[B13] Scottish Intercollegiate Guidelines Network: A guidelines developers' handbook http://www.sign.ac.uk/guidelines/fulltext/50/index.html

[B14] Centre for Evidence-Based Medicine de Oxford. Levels of Evidence and Grades of Recommendation http://www.cebm.net/index.aspx?o=1025

[B15] Atkins D Eccles M Flottorp S Guyatt GH Henry D Hill S Liberati A O'Connell D Oxman AD Phillips B Schünemann H Edejer TT Vist GE Williams JW The GRADE working group Systems for grading the quality of evidence and the strength of recommendations I: critical appraisal of existing approaches The GRADE Working Group BMC Health Serv Res 2004 4 1 38 10.1186/1472-6963-4-38 15615589 PMC545647

[B16] Schünemann HJ Best D Vist G Oxman AD Letters, numbers symbols and words: how to communicate grades of evidence and recommendations CMAJ 2003 169 7 677 680 14517128 PMC202287

[B17] Schünemann HJ Jaeschke R Cook DJ Bria WF El-Solh AA Ernst A Fahy BF Gould MK Horan KL Krishnan JA Manthous CA Maurer JR McNicholas WT Oxman AD Rubenfeld G Turino GM Guyatt G ATS Documents Development and Implementation Committee An official ATS statement: grading the quality of evidence and strength of recommendations in ATS guidelines and recommendations Am J Respir Crit Care Med 2006 174 5 605 614 10.1164/rccm.200602-197ST 16931644

[B18] Guyat GH Oxman AD Kunz R Falck-Ytter Y Vist GE Liberati A Schünemann HJ GRADE Working Group Going from evidence to recommendations BMJ 2008 336 7652 1049 1051 10.1136/bmj.39493.646875.AE 18467413 PMC2376019

[B19] Guyatt GH Oxman AD Vist GE Kunz R Falck-Ytter Y Alonso-Coello P Schünemann HJ GRADE Working Group GRADE: an emerging consensus on rating quality of evidence and strength of recommendations BMJ 2008 336 7650 924 926 10.1136/bmj.39489.470347.AD 18436948 PMC2335261

[B20] Guyatt GH Oxman AD Kunz R Vist GE Falck-Ytter Y Schünemann HJ for the GRADE Working Group What is 'quality of evidence' and why is it important to clinicians? BMJ 2008 336 7651 995 998 10.1136/bmj.39490.551019.BE 18456631 PMC2364804

[B21] Schünemann HJ Hill SR Kakad M Vist GE Bellamy R Stockman L Wisløff TF Del Mar C Hayden F Uyeki TM Farrar J Yazdanpanah Y Zucker H Beigel J Chotpitayasunondh T Hien TT Ozbay B Sugaya N Oxman D Transparent development of the WHO rapid advice guidelines PLoS Med 2007 4 5 e119 10.1371/journal.pmed.0040119 17535099 PMC1877972

[B22] Schünemann HJ Oxman AD Brozek J Glasziou P Jaeschke R Vist GE Williams JW Jr Kunz R Craig J Montori VM Bossuyt P Guyatt GH GRADE Working Group Grading quality of evidence and strength of recommendations for diagnostic tests and strategies BMJ 2008 336 7653 1106 1110 10.1136/bmj.39500.677199.AE 18483053 PMC2386626

[B23] Cabriada-Nuño JL Domènech E Gomollón F González-Carro P González-Lara V Hinojosa J Jiménez-López CE Nos P Obrador A Panès J Saro C Varea V Lafuente R Guilera M Documento Consenso en el uso de la granulocitoaféresis en pacientes con enfermedad inflamatoria intestinal Gastroenterol Hepatol 2006 29 85 91 10.1157/13083905 16448611

[B24] Índice de actividad de la colitis ulcerosa: Índice Clínica Mayo http://www.ghcontinuada.com/cgi-bin/wdbcgi.exe/gh/utiles.colitis_mayoclinic

[B25] Gutiérrez-Ibarluzea I Arcelay A Leucocitoaféresis para el tratamiento de la enfermedad inflamatoria intestinal Informe de Revisión. Vitoria-Gasteiz. Departamento de Sanidad Gobierno Vasco 2004. Informe n° Osteba IR-04-03

[B26] GRADEpro [Computer program]. Version 3.2 for Windows 2008 Jan Brozek, Andrew Oxman, Holger Schünemann

[B27] Sands BE Sandborn WJ Feagan B Löfberg R Hibi T Wang T Gustofson LM Wong CJ Vandervoort MK Hanauer S Adacolumn Study Group A randomized double-blind, sham-controlled study of granulocyte/monocyte apheresis for active ulcerative colitis Gastroenterology 2008 135 2 400 409 10.1053/j.gastro.2008.04.023 18602921

[B28] D'Haens G Sandborn WJ Feagan BG Geboes K Hanauer SB Irvine EJ Lémann M Marteau P Rutgeerts P Schölmerich J Sutherland LR A review of Activity Indices and Efficacy End Points for Clinical Trials of Medical Therapy in Adults with Ulcerative Colitis Gastroenterology 2007 132 2 763 786 10.1053/j.gastro.2006.12.038 17258735

[B29] Sawada K Muto T Shimoyama T Satomi M Sawada T Nagawa H Hiwatashi N Asakura H Hibi T Multicenter randomized controlled trial for the treatment of ulcerative colitis with a leukocytapheresis column Curr Pharm Des 2003 9 4 307 321 10.2174/1381612033391928 12570823

[B30] Panés J Guilera M Ginard D Hinojosa J González-Carro P González-Lara V Varea V Domènech E Badia X Treatment cost of ulcerative colitis is apheresis with Adacolumn cost-effective? Dig Liver Dis 2007 39 7 617 625 10.1016/j.dld.2007.03.007 17531555

[B31] Jo Y Matsumoto T Mibu R Iida M Addition of leukocytapheress to steroid therapy: is it beneficial in recurrence of moderate-to severe ulcerative colitis? Dis Colon Rectum 2003 46 10 Suppl S3 9 14530652 10.1097/01.DCR.0000088851.79497.9B

[B32] Ricart E Esteve M Andreu M Casellas F Monfort D Sans M Oudovenko N Lafuente R Panes J Evaluation of 5 versus 10 granulocytapheresis sessions in steroid-dependent ulcerative colitis: A pilot prospective, multicenter randomized study World J Gatroenterol 2007 13 15 2193 2197 10.3748/wjg.v13.i15.2193 PMC4146843 17465500

[B33] Cabriada-Nuño J Bernal-Martínez A Hernández-Martín A Arévalo-Serna J Fernández-Prado E Aféresis leucocitaria en la inducción de la remisión y retirada de corticoides en la colitis ulcerosa corticodependiente Dial Traspl 2007 28 2 47 55

[B34] Johnson FR Özdemir S Mansfield C Mansfield C Hass S Miller DW Siegel CA Sands BE Crohn's Disease Patients' Risk-Benefit Preferences: Serious Adverse Event Risks Versus Treatment Efficacy Gastroenterology 2007 133 3 769 779 10.1053/j.gastro.2007.04.075 17628557

[B35] Atkins D Briss PA Eccles M S Flottorp S Guyatt GH Harbour RT Hill S Jaeschke R Liberati A Magrini N Mason J O'Connell D Oxman AD Phillips B Schünemann H Edejer TT Vist GE Williams JW The GRADE Working Group Systems for grading the quality of evidence and the streght of recommendations II: Pilot study of a new system BMC Health Serc Res 2005 5 1 25 10.1186/1472-6963-5-25 PMC1084246 15788089

[B36] Guyatt GH Oxman AD Kunz R Jaeschke R Helfand M Liberati A Vist GE Schünemann HJ for the GRADE working group Incorporating considerations of resources use into grading recommendations BMJ 2008 336 7654 1170 1173 10.1136/bmj.39504.506319.80 18497416 PMC2394579

[B37] Calderón C Rotaeche R Etxebarria A Marzo M Rico R Barandiaran M Gaining insight into the Clinical Practice Guideline development processes: qualitative study in a workshop to implement the GRADE proposal in Spain BMC Health Serv Res 2006 6 138 10.1186/1472-6963-6-138 17059600 PMC1626459

[B38] Domènech E Hinojosa J Esteve-Comas M Gomollón F Herrera JM Bastida G Obrador A Ruiz R Saro C Gassull MA Spanish Group for the Study of Crohn's Disease and Ulcerative Colitis (GETECCU) Granulocyte apheresis in steroid-dependent inflammatory bowel disease: a prospective open, pilot study Aliment Pharmacol Ther 2004 20 1347 1352 10.1111/j.1365-2036.2004.02288.x 15606397

[B39] Muratov V Lundahl J Ulfgren AK Elvin K Fehrman I Ahlborg N Ost A Hittel N Saniabadi A Löfberg R Down-regulation of interferon-gamma parallels clinical response to selective leukocyte apheresis in patients with inflammatory bowel disease: a 12 month follow-up study Int J Colorectal Dis 2006 21 493 504 10.1007/s00384-005-0069-2 16538495

[B40] Efficacy Study of Granulocytapheresis Plus steroids vs Steroids alone in active steroid dependent ulcerative colitis (ATICCA) http://clinicaltrials.gov/ct2/show/NCT00702611

[B41] Stange EF Travis S Vermeire S Reinisch W Geboes K Barakauskiene A Feakins R Fléjou JF Herfarth H Hommes DW Kupcinskas L Lakatos PL Mantzaris GJ Schreiber S Villanacci V Warren BF for the European Crohn's and Colitis Organisation (ECCO) European evidence-based Consensus on the diagnosis and management of ulcerative colitis: definitions and diagnosis JCC 2008 2 1 23 10.1016/j.crohns.2007.11.001 21172194

[B42] Abreu MT Plevy S Sands BE Weinstein R Selective Leukocyte Apheresis for the Treatment of Inflammatory Bowel disease J Clin Gastroenterol 2008 41 10 874 888 10.1097/MCG.0b013e3180479435 18090155

